# Autoregulation of H^+^/lactate efflux prevents monocarboxylate transport (MCT) inhibitors from reducing glycolytic lactic acid production

**DOI:** 10.1038/s41416-022-01910-7

**Published:** 2022-07-15

**Authors:** Wiktoria Blaszczak, Hannah Williams, Pawel Swietach

**Affiliations:** Department of Physiology, Anatomy & Genetics, University of Oxford, Sherrington Building, Parks Road, OX1 3PT Oxford, UK

**Keywords:** Metabolic pathways, Drug development

## Abstract

**Background:**

Pharmacological inhibition of membrane transporters is expected to reduce the flow of solutes, unless flux is restored (i.e., autoregulated) through a compensatory increase in the transmembrane driving force. Drugs acting on monocarboxylate transporters (MCTs) have been developed to disrupt glycolytic metabolism, but autoregulation would render such interventions ineffective. We evaluated whether small-molecule MCT inhibitors reduce cellular H^+^/lactate production.

**Methods:**

Cellular assays measured the relationship between MCT activity (expressed as membrane H^+^/lactate permeability; P_HLac_) and lactic acid production (inferred from H^+^ and lactate excretion; J_HLac_) in a panel of pancreatic ductal adenocarcinoma (PDAC) cells spanning a range of glycolytic phenotype.

**Results:**

MCT activity did not correlate with lactic acid production, indicating that it is not set by membrane permeability properties. MCT inhibitors did not proportionately reduce J_HLac_ because of a compensatory increase in the transmembrane [lactate] driving force. J_HLac_ was largely insensitive to [lactate], therefore its cytoplasmic build-up upon MCT inhibition does not hinder glycolytic production. Extracellular acidity, an MCT inhibitor, reduced J_HLac_ but this was via cytoplasmic acidification blocking glycolytic enzymes.

**Conclusions:**

We provide mathematically verified evidence that pharmacological and physiological modulators of MCTs cannot proportionately reduce lactic acid production because of the stabilising effect of autoregulation on overall flux.

## Introduction

Cancer cells harness considerable energy from glycolytic metabolism, and in doing so, generate large quantities of lactic acid even under aerobic conditions (the Warburg effect). As the terminal step in this process, H^+^-lactate export by monocarboxylate transporters (MCTs) is considered an attractive target for controlling cancer metabolism therapeutically [1–3] for at least two reasons. Firstly, MCT isoforms, notably MCT1 (*SLC16A1*) and MCT4 (*SLC16A3*), conduct the vast majority of lactic acid efflux from cells [4], and blocking these is intuitively predicted to impact glycolytic metabolism. Secondly, surface-expressed MCTs are accessible to a range of chemical modulators [5, 6]. These considerations led to the assertion that MCT blockers, by reducing membrane H^+^/lactate permeability, also proportionately inhibit glycolytic lactic acid production, a key component of tumour metabolism. This presumed relationship between H^+^/lactate permeability and production has underpinned the rationale for developing novel MCT inhibitors [7–10] and studying physiological MCT modulation [11–13].

To investigate the relationship between MCT inhibition and the cell’s lactic acid output, it is important to consider the factors determining flux. By definition, net metabolic production of lactic acid inside cells (J_HLac_) must be in balance with transmembrane removal through MCTs (as H^+^/lactate) and the lipid bilayer (as lactic acid). As shown in the Appendix, this can be described mathematically as the product of overall membrane permeability and driving force defined in terms of the intracellular (i) and extracellular (e) concentrations:

1 $${{{{{\mathrm{J}}}}}}_{{{{{{\mathrm{HLac}}}}}}} = {{{{{\mathrm{P}}}}}}_{{{{{{\mathrm{HLac}}}}}}} \times \left( {\left[ {{{{{{\mathrm{H}}}}}}^ + } \right]_{{{{{\mathrm{i}}}}}} \times \left[ {{{{{{\mathrm{lactate}}}}}}} \right]_{{{{{\mathrm{i}}}}}}\,-\,\left[ {{{{{{\mathrm{H}}}}}}^ + } \right]_{{{{{\mathrm{e}}}}}} \times \left[ {{{{{{\mathrm{lactate}}}}}}} \right]_{{{{{\mathrm{e}}}}}}} \right)$$

**Fig. 1:**
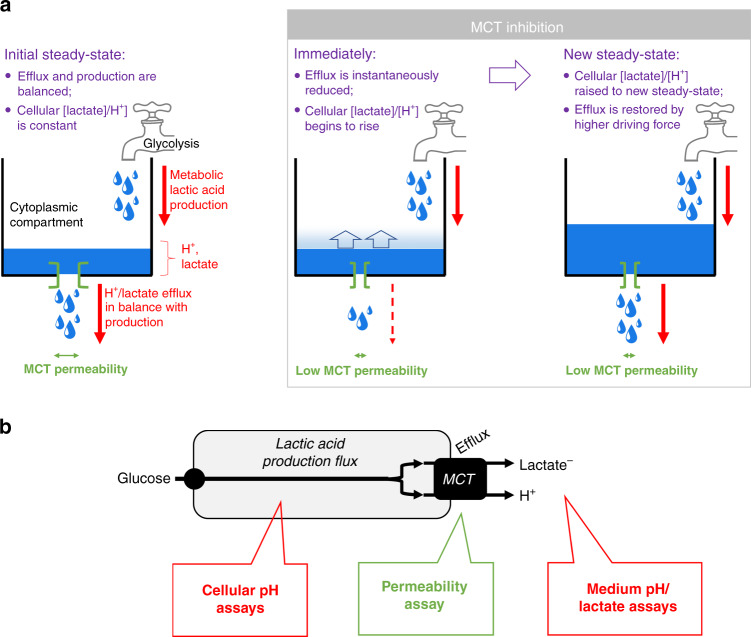
In a system that auto-regulates efflux, pharmacological inhibition of a transporter does not necessarily reduce the carried flux. **a** By definition, net metabolic production of lactic acid (represented by tap) is equal to H^+^/lactate efflux across the membrane, largely mediated by surface-expressed MCTs (represented by green vent). The level of H^+^/lactate in the cytoplasmic compartment is set by the production flux and permeability. A drug-evoked reduction in membrane permeability will initially reduce efflux. This results in a build-up of lactate/H^+^ in the cell, which increases the driving force for efflux. A new steady-state is attained, at which efflux is restored, despite the lower permeability. For this to take place, the build-up of H^+^/lactate must not block production, the ultimate driver of lactic acid release from cells. **b** Schematic of the relationship between glycolytic flux and MCT-dependent H^+^/lactate efflux, indicating the variables interrogated by assays. Flux (typically in units of mM per min) described the rate at which a solute is produced (e.g., by glycolysis) or transported (e.g., by MCTs). Permeability is a property of the cell membranes that measures how easily solutes can cross (e.g., aboard MCTs). Pharmacological drugs are often designed to influence flux by interrupting permeability, but this may not hold true if the system shows autoregulation of flux.

Here, P_HLac_ describes the total conductance of surface-expressed MCTs to H^+^ and lactate ions, after considering all relevant influences (e.g., allosteric actions and affinity) plus a lesser contribution from MCT-independent permeability across the lipid bilayer. MCT inhibitors act directly to reduce P_HLac_ by interrupting the transport cycle; however, a reduction in P_HLac_ may not necessarily decrease J_HLac_ if the driving force increases in a compensatory manner as a result of the solute build-up in the cytoplasm. This scenario is a case of autoregulation because flux remains constant irrespective of membrane permeability properties (Fig. 1a). The compensatory increase in driving force could take the form of cytoplasmic lactate and/or H^+^ ions accumulation, provided this does not hinder further metabolic production. Intracellular H^+^ ions are known to block glycolytic enzymes [14] (e.g., phosphofructokinase-1 [15, 16]), thus cytoplasmic [H^+^] build-up is unlikely to provide the necessary increase in driving force. However, cytoplasmic [lactate] accumulation could plausibly boost the driving force for MCT-operated efflux without blocking H^+^/lactate production. Since enzyme-dependent steps in glycolysis are near-equilibrium and have spare capacity to adjust to changes in load [17], autoregulation is plausible but not yet verified. An appraisal of autoregulation is important because it defines the scope of MCT inhibitors to influence lactic acid production. Biologically, the rationale for autoregulation would be in stabilising energy flows and preventing isolated changes (e.g., in MCT activity) from disrupting metabolic flows [18]. Indeed, there are precedents where a change to permeability does not proportionately alter flux. In cardiomyocytes, for example, inhibitors (or activators) of ryanodine receptor-type calcium channels cannot change calcium release from the sarcoplasmic reticulum store because of compensatory adjustments to the driving force [19]. If H^+^/lactate efflux from cells were similarly autoregulated, then MCT modulators would be ineffective in altering lactic acid production directly. An appraisal of autoregulation is therefore warranted to assess the feasibility of influencing cancer metabolism by targeting MCT pharmacologically.

To test for autoregulation, we used innovative cell-based approaches that interrogate P_HLac_ and J_HLac_ independently on the same biological material (Fig. 1b). For this, we used six pancreatic ductal adenocarcinoma (PDAC) cell lines that span a range of glycolytic phenotype. It was important that the biological responses were probed over a period long enough to engage autoregulation (i.e., several hours) because measurements of short-term responses (e.g., by Seahorse XF Analyzers) are likely to miss the autoregulatory phenomenon. Autoregulation was tested against its characteristic features, namely (i) lack of correlation between P_HLac_ and J_HLac_, (ii) absence of a sustained effect of MCT inhibitors on lactic acid release from cells and (iii) evidence that driving force can be changed in a manner that does not hinder lactic acid production. We find that H^+^/lactate efflux meets these criteria and conclude that drugs targeting MCTs are not effective in blocking lactate production over a realistic dosage range.

## Methods

### Cell culture and media

Cells were cultured in RPMI (Sigma-Aldrich, R0883) supplemented with 10% FBS, 1% penicillin–streptomycin mixture, 1% GlutaMAX (35050-038, Gibco, Waltham, MA, USA), 1% sodium pyruvate (11360-039, Gibco, Waltham, MA, USA). In the case of media incubated in 5% CO_2_, the appropriate [HCO_3_^−^] was determined empirically to equilibrate at pH 7.4. Other media were buffered with non-volatile buffers, such as HEPES or MES, and titrated to the desired pH with HCl or NaOH. Equimolar HEPES and MES provided near-constant pH buffering over the biological pH range. NaCl was added to maintain osmolarity constant [20]. For some experiments, cells were incubated in with 1 mM dimethyloxalylglycine (DMOG; 4408, Tocris) for 48 h to stabilise HIF signalling. Cell lines were authenticated by AuthentiCell service provided by the European Collection of Authenticated Cell Cultures (a part of UK Health Security Agency).

### Superfusion

Cells were seeded at 70,000 per well in 4-chamber Nunc Lab-Tek slides (734-2060, ThermoScientific). Experiments involving superfusion were performed on a Leica LCS confocal microscope to image fluorescence. Most experiments measured intracellular pH using cSNARF1 (17 μM for 10 min; C1272 ThermoFisher), excited at 514 nm and collected at 580/640 nm. The ratio was calibrated to units of pH using a calibration curve determined in separate experiments using the nigericin technique [21]. Solutions were delivered by a system of tubes operated by a peristaltic pump, with a two-level solution switcher to alternate between one of two superfusates heated to 37 °C. Excess solution was drawn by a vacuum pump to ensure laminar flow. Solutions were based on normal Tyrode containing 4.5 mM KCl, 1 mM CaCl_2_, 1 mM MgCl_2_, 11 mM glucose, 10 mM HEPES, 10 mM MES and 130 mM NaCl, titrated to the desired pH (5.7–7.7) using HCl or NaOH.

### Rapid solution switching

cSNARF1-loaded PDAC cells seeded in Lab-Tek chambers were superfused with normal Tyrode. A dual microperfusion device was manipulated into the field of view, 100 microns from the cells of interest. Switching a system of valves alternated between the two microstreams, driven by gravity flow. The speed of solution switching is 25 ms [22]. To evoke MCT activity, the first microstream contained 30 mM l-lactate at pH 7.4 (osmotically compensated with NaCl) and the second was lactate-free titrated to a particular test pH (5.7–7.7). Cells were equilibrated with the lactate microstream for 5 min to load cytoplasm with MCT substrate. MCT activity could therefore be mapped as a function of pHe from the initial rate of pHi change [23]. This protocol keeps the thermodynamic driving force constant, whilst allowing pHe to change (note, [H^+^]_e_×[lactate]_e_ is zero under superfusion with lactate-free microstream). For measurements in the presence of the MCT1 inhibitor SR13800, the MCT1/2 inhibitor AR-C155858 (1 µM; 4960, Tocris [24]) or the MCT4/1 inhibitor syrosingopine (1 µM or 10 µM; SML1908, Merck) drug was added to both microstreams to ensure adequate access time to the inhibitory site. For the single-cell glycolytic rate assay, the first microstream contained lactate-free normal Tyrode and the second was supplemented with 2 mM α-cyano-4-hydroxycinnamate (CHC; S8612, Selleckchem) to block lactic acid efflux by MCT. Both microstreams contained 30 μM 5-*N,N*-dimethylamiloride to block acid extrusion by Na^+^/H^+^ exchanger-1, the dominant pH regulator. For assaying the rate of intracellular acidification, the first microstream contained normal Tyrode and the second was titrated to either pH 5.7 or 6.6.

### Well-based fluorimetric assay of lactic acid production

The time course of medium acidification was used to gauge lactic acid production. Cells were seeded onto a 96-well plate at high density (55,000/well) and left to adhere for 24 h in standard RPMI. Cells were then washed in PBS twice. Replacing with 100 µL medium buffered with 2 mM HEPES/2 mM MES, titrated to a target pH and containing cSNARF1-dextran (0.083 mg/ml, D3304, ThermoFisher Scientific) initiated the measurement. In some experiments, media were supplemented with 10 mM or 20 mM l-lactate, or 5 mM of acetate, propionate, butyrate, phosphate or imidazole, and then re-titrated to the desired pH. The plate was placed immediately in a Biotek Cytation 5 plate reader pre-heated to 37 °C, recording fluorescence at 590 nm and 640 nm excited by 555 nm light every 30 min. The combination of low buffering and high confluency enabled the system to acidify. Control wells containing no cells were used to offset measurements. To calculate cumulative acid production, the stepwise pHe change was multiplied by medium buffering capacity and integrated.

### Well-based fluorimetric assay of lactic acid production and respiratory rate

This dual-dye fluorimetric assay was based on a recently published method [25].

### Biochemical lactate assay

After a period of incubation, the medium was collected and frozen at −80 °C to deactivate l-lactate dehydrogenase prior to [lactate] measurements on a YSI 2500 Biochemistry Analyzer (525000, Xylem) or ABX Pentra C400 analyzer (Horiba).

### Western blotting

Samples were sonicated, denatured by BME in Laemmli buffer (1:20). Gels were prepared with 15% acrylamide. The primary antibodies were raised against MCT1 (1:500, Millipore Cat# AB3538P, RRID:AB_2189203, rabbit), MCT2 (1:4000, Proteintech Cat# 20355-1-AP, AB_2878680, rabbit) MCT4 (Millipore Cat# AB3316P, RRID:AB_2189344, rabbit), HIF1α (1:1000, BD Biosciences Cat # 610958, RRID:AB_398271, mouse), HRP-conjugated GAPDH 1:6000 (Proteintech Cat# HRP-60004, RRID:AB_2737588). The secondary antibody was HRP-goat anti-rabbit (1:4000, 6556120, Invitrogen). To detect total protein lactylation samples were collected and denatured as described previously and separated on 12.5% polyacrylamide gel. The primary antibody was raised against l-lactyl-lysine (1:1000, PTM-1401, PTM-Biolabs, rabbit), and secondary HRP-conjugated and raised against β-actin (1:5000, Proteintech, mouse).

### Seahorse XF assays

Media for ECAR and OCR measurements on the Seahorse XFe96 Analyzer (Agilent) were prepared with 2 mM HEPES and 2 mM MES to provide light buffering that is constant over the biological pH range. Media were titrated to pH 7.4 but not supplemented with FBS or antibiotics. Consecutive injections of 0.5 N HCl and 0.4 M NaOH produced a decrease and then a recovery of pH. PDAC cells were seeded onto a 96-well plate 24 h prior to experiments in a standard RPMI-based medium at a density of 25k/well. Cells were washed twice with PBS before adding 100 µL of the final medium. Initial and post-injection values of ECAR and OCR were recorded, confirmed for reversibility and normalised to readings at pH 7.4.

### High-throughput pH imaging

Cells were plated in 96-well plates and loaded with cSNARF1 (used to measure pHi) and Hoechst (used to identify nuclei for cell segmentation) for 10 min. Media were then replaced with formulations buffered with 10 mM HEPES and 10 mM MES, titrated to a pH between 5.5 and 7.7. After allowing 10 min for equilibration, images were taken using the Biotek Cytation 5 at 37 °C [20].

### Statistical analysis

The number of observations is reported as “n/N”, indicating the number of cells or wells and the number of biological repeats yielded from separate plating and passage. Additional tests used are listed in corresponding figure legends. At least three biological repeats were performed (i.e., from an independent cell passage), with at least three technical repeats each (i.e., concurrent measurements from same yield of cells, but treated separately to drugs, e.g., in a separate well). All *t* tests were two-sided. Summary statistics are shown as mean ± SEM, unless stated otherwise. Significance level for all tests was 5%.

See supplement for further methods.

## Results

### Across different PDAC lines, MCT-facilitated H^+^/lactate permeability does not correlate with lactic acid production

**Fig. 2:**
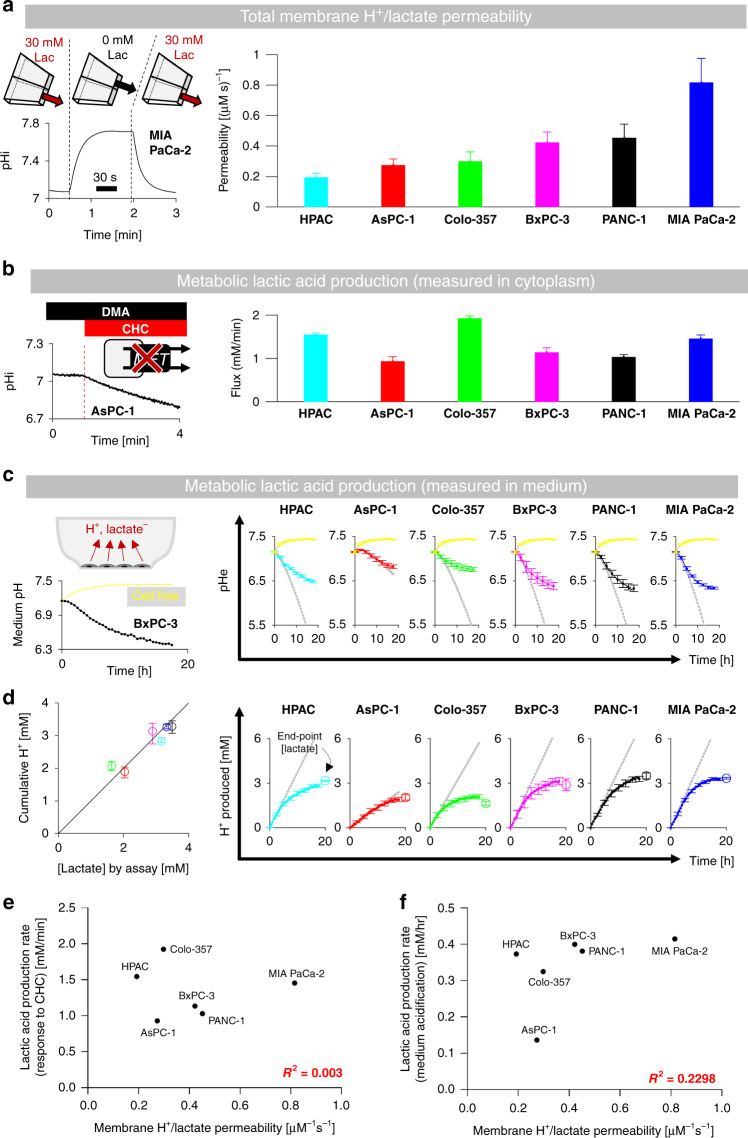
Membrane H+/lactate permeability does not correlate with lactic acid production. **a** Measuring MCT-dependent H^+^/lactate permeability (P_HLac_) using rapid solution switcher alternately releasing 30 mM lactate-containing and lactate-free microstreams. Exemplar pHi time course (cSNARF1). P_HLac_ in six PDAC cell lines (*n* = 2500–3500 cells/*N* = 4–6 platings). **b** Lactic acid production rate inferred from the immediate effect on pHi of MCT blockade with α-cyano-4-hydroxycinnamate (CHC; 2 mM) (*n* = 500/*N* = 2). **c** Lactic acid production measured from medium acidification (cSNARF1-dextran) and end-point lactate by biochemical assay (*n* = 9/*N* = 3). Yellow traces are cell-free wells; grey lines indicate the initial rate. **d** Concordance between lactate and H^+^ production in media (Pearson’s *R* = 0.93; *P* = 0.008). Medium pH time courses converted to cumulative acid production, also showing endpoint [lactate] measured biochemically. **e**, **f** Lack of significant correlation between P_HLac_ and lactic acid production measured by the two assays (*R*^2^ is the Pearson correlation coefficient).

The relationship between MCT-facilitated membrane H^+^/lactate permeability (P_HLac_) and metabolic lactic acid production (J_HLac_) was first studied using cellular assays performed on a panel of six PDAC cell lines. If the membrane’s conductance to H^+^/lactate, facilitated by surface-expressed MCTs, was rate-limiting for lactic acid release from cells, a linear P_HLac_-J_HLac_ relationship would be expected across different cell lines. Membrane permeability was measured by manipulating the extracellular concentration of lactate to drive net efflux across MCTs. For this approach to be accurate, the solution change must be rapid, so that permeability is not underestimated because of a slow-mixing artefact. To avoid this common problem, extracellular solutions were changed by means of an ultra-rapid solution switcher [22]. This device alternately released a microstream containing 30 mM lactate to load cells with MCT substrate, and a lactate-free microstream to activate H^+^-lactate efflux. Lactic acid efflux was calculated from the rate of intracellular alkalisation (measured with cSNARF fluorescence; Fig. 2a), multiplied by the intrinsic buffering capacity (Supplementary Fig. S1). Flux was then converted to P_HLac_ using known values of [H^+^]_e_, [lactate]_e_, [H^+^]_i_ and an estimate of [lactate]_i_ from Eq. (1). Uniquely, this technique can exchange solutions in under 30 ms, ensuring that P_HLac_ is not underestimated by slow mixing, a concern with most conventional superfusion systems. P_HLac_ measured in the six PDAC cells is shown in Fig. 2a, ranked by magnitude; permeability was four times higher in MIA PaCa-2 cells compared to HPAC cells. In all cases, measured permeability was at least two orders of magnitude higher than MCT-independent permeability (see Appendix).

The rate of lactic acid production by cells (J_HLac_) was measured by two independent methods. The first approach inferred J_HLac_ from the intracellular pH (pHi) response to an abrupt and broad-spectrum inhibition of MCTs with α-cyano-4-hydroxycinnamate (CHC; 2 mM) delivered by the rapid solution switcher. Abrupt blockade of MCT traps endogenously produced lactic acid in the cytoplasm, causing an immediate cytoplasmic acidification that, when multiplied by intrinsic buffering capacity, measures J_HLac_ (Fig. 2B). To inactivate H^+^-handling transporters, which would normally restore pHi to normal, superfusates were CO_2_/HCO_3_^−^ free to inactivate HCO_3_^−^-dependent pHi regulators (e.g., Na^+^-HCO_3_^−^ cotransporters) and contained 30 μM DMA to block Na^+^/H^+^ exchanger activity, the main residual pHi-regulator present under a HEPES-buffered regime. As a secondary effect, CHC may block monocarboxylate (e.g., pyruvate) uptake into the mitochondria, thereby diverting fluxes to glycolysis and, potentially, over-estimate of glycolytic rate. To test the extent to which this may occur, experiments were repeated under conditions where the metabolic substrate (glucose) is replaced with galactose. This switch attenuates lactic acid production [25], as well as the CHC-response by tenfold (Supplementary Fig. S2). This finding indicates that the flux response to CHC provides a fair measure of glycolytic rate.

The second approach interrogated J_HLac_ from the initial rate of medium acidification arising from lactic acid release. To initiate medium acidification, standard culture media were replaced with a lightly buffered formulation (2 mM HEPES, 2 mM MES), which ensures a resolvable pH response to glycolytic production. A limitation of this method is that it reports the ensemble rate for the population of cells and is therefore sensitive to seeding density. Medium pH was measured from cSNARF1-dextran fluorescence (Fig. 2c) and converted to a time course of acid production by integrating the product of medium pH change and its buffering capacity (Fig. 2d). This approach was verified to provide an accurate readout of lactic acid production by the excellent agreement between cumulative acid production and [lactate] at the experimental endpoint. The initial rate of acidification was measured from the slope of the acid-production time course, at which point the cell density and medium conditions were the same in all experiments.

J_HLac_ measured using either approach did not correlate with P_HLac_ (Fig. 2e, f). The absence of a linear P_HLac_-J_HLac_ correlation is consistent with flux autoregulation and must reflect substantial differences in driving force between cells of different lines. The lack of correlation also indicates that a measurement of membrane H^+^/lactate permeability (or equivalent, e.g., *SLC16* expression) cannot predict glycolytic lactic acid production.

### Pharmacological MCT inhibition does not proportionately reduce lactic acid release from cells

**Fig. 3:**
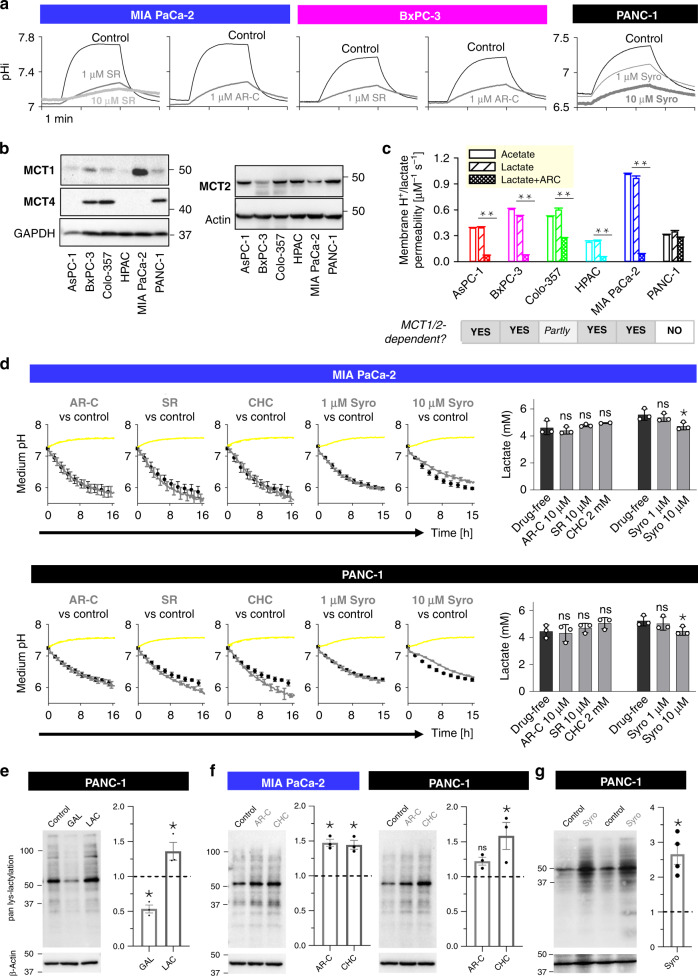
Pharmacological MCT inhibitors do not block lactic acid production. **a** Confirmation of the inhibitory effect of SR13800 (SR), AR-C155858 (AR-C) and syrosingopine (Syro) on H^+^/lactate permeability. **b** Western blot showing expression of MCT1, MCT2 and MCT4. **c** Membrane permeability measured for H^+^/lactate in the presence and absence of MCT1/MCT2 inhibitor AR-C (1 µM), and compared to H^+^/acetate (*n* = 500–1000/*N* = 3). **P < 0.01, multiple comparisons *t* test. **d** Medium acidification (cSNARF1-dextran) in presence of AR-C (10 µM), SR (10 µM) or CHC (2 mM). Cumulative acid production and endpoint [lactate] are unaffected by MCT inhibitors (*n* = 3 repeats each; not significant; one-way ANOVA) The data are plotted as mean ± SD. **e** Western blot showing response of protein lactylation in media containing galactose instead of glucose (GAL) or media supplemented with lactate (LAC), and densitometric analysis. **f** Lactylation increases after treatment with 10 µM AR-C (*n* = 3) and 2 mM CHC (*n* = 3) and (**g**) 10 µM Syro (*n* = 4). *P < 0.05, repeated measures ANOVA). Densitometric analyses were normalised to controls (dashed lines).

Flux through a system that auto-regulates is not expected to decrease in proportion to a reduction in membrane permeability. To test if lactic acid production auto-regulates, its response to MCT inhibition was tested. For this, drugs used previously to block MCTs were investigated at appropriate concentrations (1 μM AR-C155858 [9], 1 μM SR13800 [26, 27], 10 μM syrosingopine [9], >1 mM CHC [28]). The pharmacological activity of the MCT1/2 inhibitor AR-C155858, MCT1 inhibitor SR13800 and MCT1/4 inhibitor syrosingopine was confirmed by the powerful inhibitory effect on ensemble P_HLac_ (Fig. 3a). SR13800 at 1 μM reduced P_HLac_ by 91% in MIA PaCa-2 cells and 85% in BxPC-3 cells. AR-C155858 (1 μM) reduced P_HLac_ by 96% in MIA PaCa-2 cells and 83% in BxPC-3 cells. In MCT4-expressing PANC-1 cells, syrosingopine reduced P_HLac_ by 51% at 1 μM and by 69% at 10 μM. It is noteworthy that the higher dose produced intracellular acidification which should be considered when interpreting the drug’s action. The MCT-blocking potency of CHC is well established and confirmed earlier (Fig. 2b). Across the six PDAC cells tested, the identity of isoforms underpinning MCT activity was determined by western blotting for MCT1, MCT2 and MCT4 (Fig. 3b) and from the sensitivity of P_HLac_ to AR-C155858 (Fig. 3c). Patterns of MCT expression were consistent with previous studies [29–31]. Overall, P_HLac_ was dominated by MCT1 in MIA PaCa-2 cells whereas MCT4 dominated activity in PANC-1 cells, therefore these two highly glycolytic lines were selected for further studies.

The effect of MCT inhibitors on lactic acid release from cells was inferred from real-time measurements of medium acidification and verified by end-point biochemical lactate assays (Fig. 3d). To ensure that the pharmacological disruption to P_HLac_ had sufficient time to implement any compensatory responses (e.g., through driving force), the time course was measured over 16 h. Despite an inhibitory effect on P_HLac_, AR-C155858 (10 μM), SR13800 (10 μM), CHC (2 mM) and syrosignopine (10 μM) did not proportionally reduce lactic acid production in MIA PaCa-2 or PANC-1 cells. There was also no significant effect of these drugs on end-point glucose levels (Supplementary Fig. S3). At the higher concentration of 10 μM, syrosingopine produced a very small (10%) decrease in lactic acid production, but this was not proportional to its effect on P_HLac_, and may relate to its effect on acidifying cytoplasm, instead. The effect of SR13800 was studied further using a fluorimetric assay that measures acid production (glycolysis) and oxygen consumption (respiration) simultaneously (Supplementary Fig. S4). Again, this showed no substantial consequences of pharmacological MCT inhibition on glycolytic metabolism or respiratory rate.

The apparent constancy of J_HLac_, despite P_HLac_ falling by as much as 90%, must be explained in terms of a steeper driving force, i.e., greater cytoplasmic lactate and/or H^+^ accumulation. Owing to the cell’s powerful pH-regulatory apparatus, substantial [H^+^]_i_ changes are unlikely; however, a cytoplasmic build-up of lactate is plausible. Evidence for stable lactate build-up in cells with pharmacologically inhibited MCT was sought by western blotting for lactylated lysines after 16 h of incubation in lightly buffered medium. This method can identify whether cells experienced a period of elevated cytoplasmic [lactate] using a biomarker that is relatively stable for sample processing. The use of lactate sensors, such as FRET probes, is less suitable for this purpose, as the protocol would require cells to be handled for imaging, during which any cytoplasmic lactate accumulation may wash away. To confirm the adequate resolving power of lactylation, experiments were performed in media where glucose was replaced with galactose (negative control) or in glucose-containing media supplemented with lactate (positive control). As expected, lactylation of proteins, particularly near 55 kDa, was reduced in galactose and increased with lactate supplementation (Fig. 3e). AR-C155858 raised protein lactylation in MCT1-dependent MIA PaCa-2, and broad-spectrum inhibition by CHC increased lactylation in both PANC-1 and MIA PaCa-2 lines (Fig. 3f). In MCT4-dependent PANC-1 cells, 10 μM syrosingopine also raised protein lactylation (Fig. 3g). In summary, MCT inhibitors at concentrations that reduce membrane H^+^/lactate permeability do not proportionately reduce lactic acid production, gauged in terms of medium acidification and lactate production. This discordance supports the notion that lactic acid efflux across the surface membrane is autoregulated because it can remain constant even at reduced permeability.

### Lactic acid production is relatively insensitive to [lactate] but strongly inhibited at low pH

**Fig. 4:**
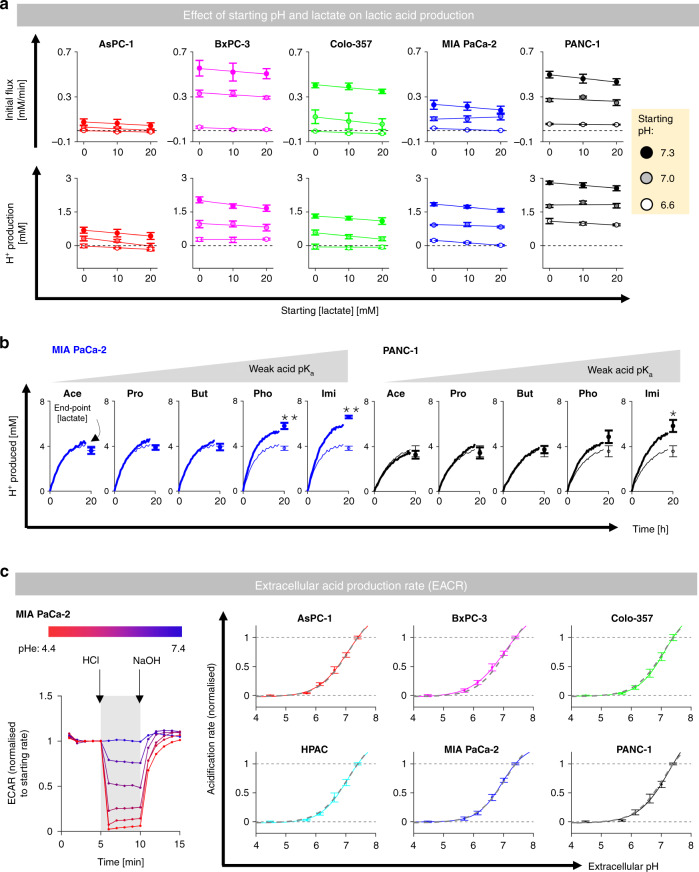
Lactic acid production is strongly influenced by pH but not [lactate]. **a** Fluorimetric assay for medium acidification (cSNARF1-dextran). Starting medium pH and [lactate] were varied (*n* = 12/*N* = 4). Two-way ANOVA: *P* < 0.001 for effect of pH, *P* > 0.05 for effect of lactate. **b** Acid production into media supplemented with weak acids of increasing pK_a_ (thick line: 5 mM of Ace-acetate, Pro-propionate, But-butyrate, Pho-phosphate, Imi-imidazole), compared to controls (thin line). Error bars not shown for clarity. Endpoint [lactate] measured by biochemical assay (*n* = 12/*N* = 4). Significant effect of buffer tested by *t* test: *P < 0.05; **P < 0.01. **c** Glycolytic production of lactic acid inferred from the extracellular acidification rate (ECAR; Seahorse) in response to reducing pH from 7.4 with HCl injection, and then restoring pH to 7.4 with NaOH injection (*n* = 8/*N* = 2). Exemplar time course shows ECAR normalised to initial rate (i.e., prior to first pH change). Grey lines show pHe-ECAR curve averaged for all six lines.

In order for efflux to remain constant under reduced permeability, the build-up of cytoplasmic solutes cannot block production. This was tested by measuring the time course of acidification of media titrated over a range of starting pH (6.6–7.3) and lactate concentration (0–20 mM). These modified initial conditions load the intra- and extracellular compartments with H^+^ and/or lactate ions, revealing any inhibitory actions on glycolytic metabolism. The rate of medium acidification trended to be only mildly sensitive to starting [lactate], but decreased sharply and significantly at lower pH (Fig. 4a). Similarly, total acid output from cells was strongly sensitive to starting pH, but only weakly reduced with higher [lactate]. These findings indicate that a cytoplasmic build-up of lactate will not block glycolysis to a significant degree, at least compared to the effect of pH. In the context of MCT inhibitors, this metabolic insensitivity to lactate would permit a steeper driving force to compensate for reduced permeability, thereby enabling autoregulation to take place.

The observation that exogenously applied [lactate] does not affect endogenous lactic acid production can be explained in terms of the transmembrane gradient. Supplementing media with lactate raises intra- and extracellular [lactate] in tandem. The inhibitory effect of reduced pH is well documented, although it is debated whether this is a consequence of MCT inhibition by extracellular H^+^ ions or glycolytic inhibition by intracellular H^+^ ions. This distinction is critical in the context of autoregulation because any form of direct MCT inhibition is not expected to block overall flux. Thus, establishing the mechanism for acid inhibition of lactic acid production is another test of autoregulation.

Consistent with an inhibitory action of H^+^ ions, lactic acid production could be dysinhibited using pH buffers, with the strongest effect attained with substances of high pK_a_ (Fig. 4b). Strikingly, a high concentration of HEPES buffer accelerated glycolytic rate to a degree that reduced respiratory demand (Supplementary Fig. S5A). The linearity between medium buffering power and lactic acid production may indicate extracellular pH (pHe) as an apparent regulator of glycolytic rate (Supplementary Fig. S5B), ostensibly acting via MCT inhibition. This inference contradicts autoregulation and merited further investigation. To test if this pHe effect is apparent on a more acute timescale, the extracellular acidification rate (ECAR) was measured by Seahorse assay in response to rapid shifts to extracellular pH (pHe) attained by HCl/NaOH injections to lightly buffered media (2 mM HEPES and 2 mM MES). The injected amounts of HCl and NaOH were determined empirically to reduce pHe from 7.4 to a test level (down to pH 4.5), and then return to 7.4. Oxygen consumption rate (OCR) was measured in parallel. In all six PDAC lines, ECAR was profoundly inhibited at low pHe (Fig. 4c), whereas OCR was remarkably insensitive (Supplementary Fig. S6).

To test if the ECAR result is a manifestation of pH-sensitive glucose transport, cellular uptake of the fluorescent derivative NBDG was measured. NBDG was loaded into cells for 15 min over a range of pHe, followed by rapid washout to clear extracellular dye. NBDG uptake, imaged in cells identified by CellTracker Red signal, showed no clear pHe-sensitivity (Supplementary Fig. S7). Thus, low pHe strongly inhibits lactic acid production, but not through an effect on glucose uptake or respiration. If the pHe effect on ECAR was via MCT inhibition, it would argue against autoregulation. To investigate this, the pHe-ECAR relationship was compared to the pHe-sensitivity of MCT activity.

### Low pHe reduces P_HLac_ but this does not underpin inhibition of lactic acid production

**Fig. 5:**
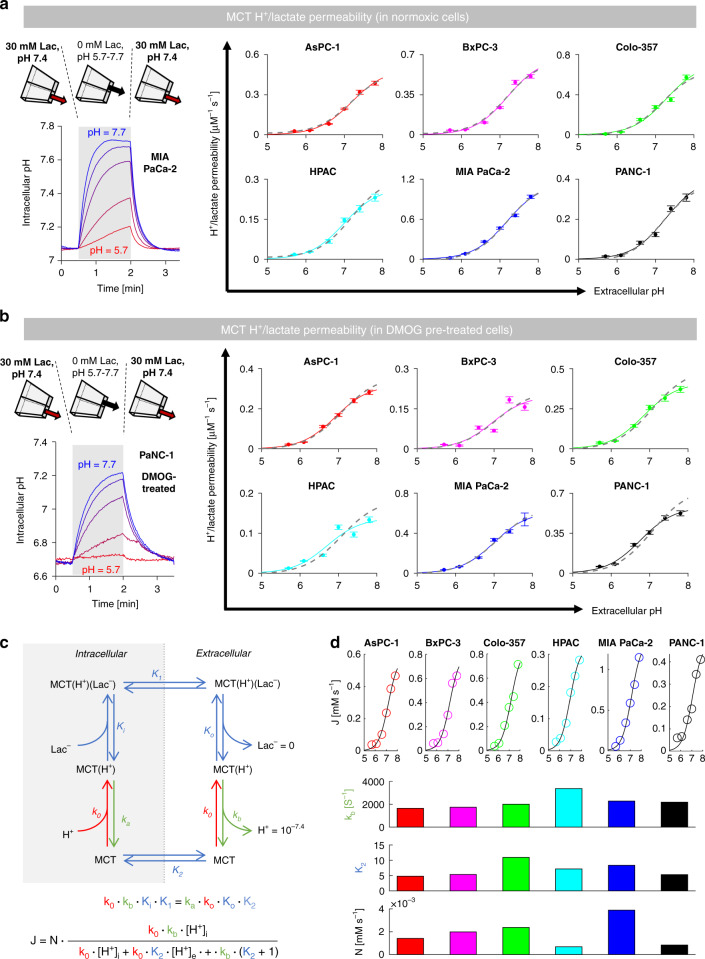
MCT-dependent H^+^/lactate permeability is sensitive to extracellular pH. **a** Protocol for mapping pHe-P_HLac_ relationship by altering pH of lactate-free superfusate (shaded). pHe-P_HLac_ relationship fitted with a non-cooperative Hill curve for six PDAC cell lines; grey lines show mean for all six lines (pK = 7.19, Hill coefficient 1.26). **b** Protocol for measuring P_HLac_ in PDAC cells after 48 h treatment with DMOG (1 mM) to induce HIF signalling. pHe-dependence fitted with a non-cooperative Hill curve for six PDAC lines. All cells could be described by the same Hill coefficient and apparent pK_a_. **c** Six-state model fit to pHe-P_HLac_ data. **d** Estimates of deprotonation rate constant k_b_ and equilibrium constant K_2_ and the copy-number of MCT proteins at the membrane.

To test how MCT responds to an acute decrease in pHe, P_HLac_ measurements were performed using the rapid switcher device, with one microstream containing 30 mM lactate at pH 7.4 and the other containing lactate-free solution titrated to a pH between 5.7 and 7.4. In all six PDAC lines, P_HLac_ was inhibited at low pHe, following a non-cooperative Hill curve (Fig. 5a). As the effect on P_HLac_ was interrogated instantaneously upon a pHe change, it likely relates to a direct inhibitory effect of extracellular H^+^ ions. Although the six PDAC lines express various combinations of MCT isoforms (see Fig. 3b), the pHe-P_HLac_ relationships were remarkably conserved even after activation of hypoxic signalling with 48 h DMOG treatment (Fig. 5b) which induces the MCT4 gene *SLC16A3* [32] (Supplementary Fig. S8), confirming a prior observation with these cell lines [33]. The process underpinning pHe-sensitivity was determined, using a six-state model of MCT (Fig. 5C) [34], to be rate-limiting deprotonation from an exofacial site of pK_a_ = 7.26 (Fig. 5d).

**Fig. 6:**
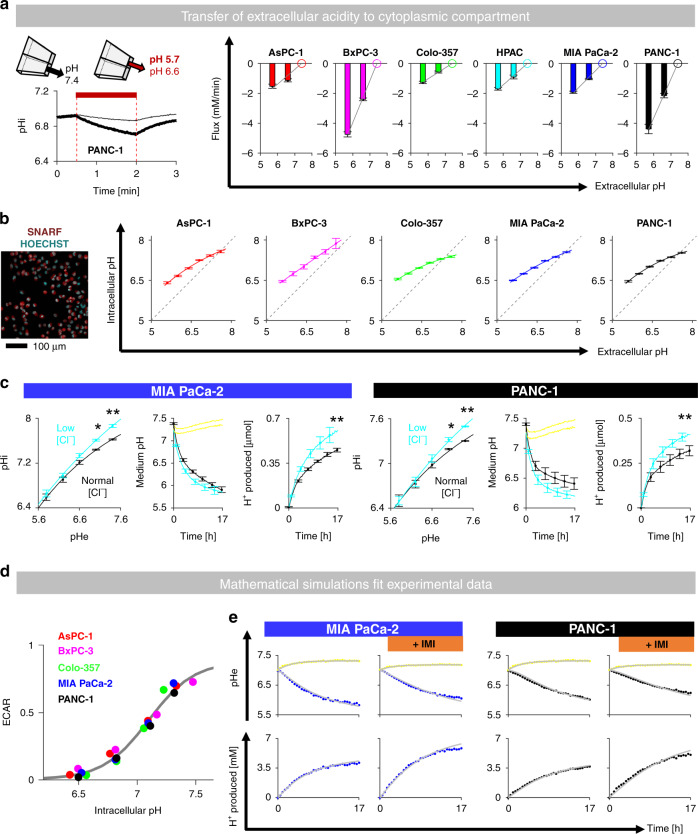
Low extracellular pH reduces lactic acid production through intracellular acidification. **a** Response of pHi to sudden extracellular acidification to pHe 6.6 or 5.7, converted to flux (*n* = 1000–3000/*N* = 3). **b** Resting pHi measured after a >10 min equilibration period at test pH (*n* = 1000–3000/*N* = 3). **c** Incubation in low-chloride medium raises pHi at constant pHe (*n* = 12/*N* = 4) and accelerates glycolysis (*n* = 12/*N* = 4; effect of low chloride: *P < 0.05, **P < 0.01 by two-way ANOVA). **d** ECAR data from five PDAC lines is described by a unique function of pHi (pKa = 7.10, Hill coefficient 2.25). **e** pHi-sensitivity is sufficient to predict the time course of medium acidification and the effect of 5 mM imidazole.

The striking resemblance between the pHe-P_HLac_ (Fig. 5a) and pHe-ECAR (Fig. 4c) relationships could be interpreted to indicate that low pHe reduces lactic acid production by reducing MCT activity. However, unlike P_HLac_ measurements reporting the immediate response to pHe, ECAR measurements are slower to acquire and during that delay, extracellular acidification could evoke intracellular responses that influence glycolysis independently of the pHe-sensitivity of P_HLac_. Indeed, the cytoplasm of cells acidifies promptly upon exposure to acidic environments, as determined in cSNARF1-loaded cells subjected to rapid solution switching (Fig. 6a). At steady-state, the relationship between pHe and pHi was curvilinear, with a slope of 0.6, as determined using a high-throughput imaging system following 10- min equilibration period in 10 mM HEPES and 10 mM MES media titrated over a range of pHe (Fig. 6b). Thus, under these experimental conditions, a 1-unit drop in pHe will, within minutes, reduce pHi by 0.6 units and hence influence glycolytic enzymes. To demonstrate that lactic acid production is directly sensitive to pHi, rather than pHe, the fluorimetric assay was performed in low-chloride medium, which alkalinises cytoplasm by inactivating acid-loading processes [35]. This pHi-selective manoeuvre significantly accelerated lactic acid production in MIA PaCa-2 and PANC-1 cells (Fig. 6c). In light of this finding, ECAR data were re-plotted as a function of pHi, and the resulting relationship was found to be highly conserved among PDAC cell lines (Fig. 6d). Crucially, application of this pHi-ECAR relationship was sufficient to predict the trajectory of medium acidification, as well as the effect of buffer supplementation, without needing to implicate a role for H^+^-sensitivity of MCT activity (Fig. 6e).

In summary, the direct inhibitory action of extracellular H^+^ ions on P_HLac_ does not underpin the reduction in glycolytic rate, which is, instead, a response to the secondary cytoplasmic acidification. This mechanism is consistent with autoregulation because lactic acid production is not rate-limited by P_HLac_.

### Simulating flux autoregulation using mathematical modelling

Mathematical modelling verified the experimental finding that P_HLac_ inhibition has only a weak effect on glycolytic lactic acid production (Supplementary Fig. S9A). Various modelling scenarios were simulated, including the in silico implementation of (i) pHi regulation, (ii) pHe-sensitivity of MCT, (iii) MCT pharmacological inhibition and (iv) pHi-sensitivity of glycolysis. Lactic acid production was decreased only in scenarios where glycolysis was pHi-sensitive. When pHi-sensitivity of glycolysis was programmatically disabled, lactic acid production was unaffected by reductions in P_HLac_ by as much as 100-fold (Supplementary Fig. S9B). Autoregulation arose from compensatory changes to the transmembrane [lactate] gradient. Thus, the results of modelling are consistent with the notion that isolated changes to P_HLac_, such as those evoked by the direct action of MCT inhibitors, do not produce a proportional effect on glycolytic output.

## Discussion

We present evidence that the efflux of glycolytically generated lactic acid across the surface membrane is autoregulated, which means that drugs acting on MCT-facilitated permeability cannot influence glycolytic production in a proportional manner. This phenomenon arises because a reduction in H^+^/lactate permeability inadvertently increases the driving force (i.e., cytoplasmic [lactate]) and restores the original magnitude of lactic acid efflux. In other words, lactic acid release from cells is ultimately driven by glycolytic production rather than rate-limited by membrane conductance. Consequently, MCT inhibitors, especially at doses attainable in vivo, are not predicted to block cellular lactic acid production, a major component of tumour metabolism. Even with maximal drug bioavailability, it is not possible to completely ablate H^+^/lactate permeability because lactic acid is able to diffuse across the lipid bilayer [36]. Our findings also caution that measurements of MCT activity or expression cannot accurately describe the glycolytic profile of cells. Indeed, any two cell lines can have dramatically different MCT activity, yet produce similar glycolytic rates, or vice versa.

Autoregulation may have been overlooked because of the design of measurements. The compensatory increase in driving force following acute MCT inhibition will not be immediate, as it requires the system to adjust to the lower permeability. For this reason, assays giving fast readouts (e.g., Seahorse), may not capture the autoregulatory correction, and give the impression that MCT blockers also reduce glycolysis. The methods implemented herein overcame this issue by tracking lactic acid release over hours, a timeline that is relevant to drug actions in vivo or under culture conditions. Various studies assessing the effects of MCT inhibitors have routinely focused on lactate influx [26, 37–39], a direction that is less relevant to most hypoxic tumour scenarios, where cells tend to be net lactate producers. We addressed this issue by studying lactate release driven by cellular metabolism. Several reports have taken lactate build-up, following MCT inhibition, as a measure of metabolic flux [26, 37–39]. Indeed, autoregulation predicts that MCT inhibition shall raise cytoplasmic lactate, but this may occur without a change in metabolic flux. The methods herein inferred flux and permeability using direct methods that provide measurement in relevant units, which can then be compared between different cells and cell lines.

Autoregulation of lactic acid flux does not preclude a genuine biological effect of MCT inhibitors on other aspects of cancer biology. We speculate that reports of reduced cell proliferation or tumour growth following MCT inhibition are a product of toxic levels of cytoplasmic lactate build-up [40] or possibly off-target effects [1, 9, 10, 41], rather than glycolytic blockade per se. In contrast to lactate, which can build-up substantially in the cytoplasm, the combination of high intracellular buffering and pHi regulation restrict the extent to which pHi is able to change, but an effect of disrupted pHi cannot be excluded as a consequence of MCT inhibitors. This may explain the acidification of cells after treatment with a high concentration of syrosingopine. If pHi does change, then this will impact glycolytic rate via the direct action of intracellular H^+^ ions on cytoplasmic enzymes [42, 43].

The action of extracellular H^+^ ions is a peculiar example of a modulator that reduces H^+^/lactate permeability and production in apparent tandem. However, inhibition of lactic acid flux is not a consequence of reduced permeability, but rather an effect of intracellular acidification on enzymes arising because of coupling between pHi and pHe. Thus, the extracellular accumulation of excreted H^+^ ions inhibits glycolysis indirectly by reducing pHi, rather than through the inhibitory effect on MCTs. The glycolysis-stimulating actions of augmented buffering [44] should therefore be interpreted in terms of the increase in pHi evoked by more alkaline environment. Although MCTs are positioned strategically to monitor the release of glycolytic end-products [45, 46], autoregulation renders this feedback ineffective. In terms of control, the condition for shutting-down glycolysis is critically low pHi, rather than a threshold chemical composition of the extracellular milieu. This highlights the importance of the pHe–pHi transfer function, and is consistent with the notion that cytoplasmic enzymes are rate-limiting for glycolysis [46].

The biological rationale for autoregulation may relate to the elimination of a potential vulnerability in the Warburg effect. Without autoregulation, fermentative metabolism would be subservient to external modulators, which often vary over a wide range [2]. Furthermore, we speculate that cells control their MCT expression to regulate intracellular [lactate] (a proxy of under-perfused, hypoxic tissues [47, 48]), rather than to control glycolytic flux.

Autoregulation of H^+^/lactate efflux in glycolytic cells draws an analogy to calcium signalling in the heart, a robust process that is notably insensitive to modulators of RyR-type calcium channels. The magnitude of calcium release is ultimately set by a pump (SERCA), and any imposed changes to RyR permeability are compensated by an adjustment to the calcium gradient [19]. In this analogy, glycolytic enzymes represent the pump, MCT activity defines permeability and lactate build-up is equivalent to the driving force.

In conclusion, we provide evidence that pharmacological targeting of MCTs does not reduce cancer cell glycolytic flux in a proportional manner, as long as pHi regulators maintain constancy of intracellular pH. We postulate that the reported biological actions of MCT inhibitors are more likely related to the build-up of lactate in the cytoplasm, rather than a change in metabolic fluxes per se.

### Supplementary information

Supplemental Mehtods and Supplemental Figures

Checklist

## Data Availability

All data are presented in the figures and supplements. Additional information and raw data are available upon reasonable request to the corresponding author.

## Appendix

The sum of lactic acid and H^+^/lactate production in a cell (J_Hlac_) must be in balance with efflux through MCTs and the lipid matrix:

$${{{\mathrm{J}}}}_{{{{\mathrm{HLac}}}}} = 	\;\rho \times {{{\mathrm{p}}}}_{{{{\mathrm{MCT}}}}} \times \left( {\left[ {{{{\mathrm{H}}}}^ + } \right]_{{{\mathrm{i}}}} \times \left[ {{{{\mathrm{lactate}}}}} \right]_{{{\mathrm{i}}}}\,-\, \left[ {{{{\mathrm{H}}}}^ + } \right]_{{{\mathrm{e}}}} \times \left[ {{{{\mathrm{lactate}}}}} \right]_{{{\mathrm{e}}}}} \right) \\ 	+ \,\rho \cdot {{{\mathrm{p}}}}_{{{{\mathrm{lipid}}}}} \times \left( {\left[ {{{{\mathrm{lactic}}}}{{{{{\mathrm{ }}}}}}\;{{{\mathrm{acid}}}}} \right]_{{{\mathrm{i}}}}\,-\, \left[ {{{{\mathrm{lactic}}}}{{{{{\mathrm{ }}}}}}\;{{{\mathrm{acid}}}}} \right]_{{{\mathrm{e}}}}} \right)$$

Where ρ is the surface area/volume ratio of the cells [µm^−1^], p_MCT_ is the MCT-dependent permeability constant to H^+^/lactate ions [µm µM^−1^ s^−1^], and p_lipid_ is the lipid matrix permeability constant to lactic acid [µm s^−1^]. The equation can be simplified by combining ρ and permeabilities (p_MCT_, p_lipid_) into conductances P_MCT_ and P_lipid_:

$${{{\mathrm{J}}}}_{{{{\mathrm{HLac}}}}} = {{{\mathrm{P}}}}_{{{{\mathrm{MCT}}}}} \times \left( {\left[ {{{{\mathrm{H}}}}^ + } \right]_{{{\mathrm{i}}}} \times \left[ {{{{\mathrm{lactate}}}}} \right]_{{{\mathrm{i}}}}\,-\,\left[ {{{{\mathrm{H}}}}^ + } \right]_{{{\mathrm{e}}}} \cdot \left[ {{{{\mathrm{lactate}}}}} \right]_{{{\mathrm{e}}}}} \right) + {{{\mathrm{P}}}}_{{{{\mathrm{lipid}}}}} \cdot \left( {\left[ {{{{\mathrm{lactic}}}}{{{{{\mathrm{ }}}}}}\;{{{\mathrm{acid}}}}} \right]_{{{\mathrm{i}}}}\,-\,\left[ {{{{\mathrm{lactic}}}}{{{{{\mathrm{ }}}}}}\;{{{\mathrm{acid}}}}} \right]_{{{\mathrm{e}}}}} \right)$$

Since lactic acid, lactate and H^+^ are in equilibrium defined by the acid dissociation constant K_Hlac_ (10^−3.86^ M):

$${{{\mathrm{K}}}}_{{{{\mathrm{HLac}}}}} = \left[ {{{{\mathrm{H}}}}^ + } \right] \cdot \left[ {{{{\mathrm{lactate}}}}} \right]/\left[ {{{{\mathrm{lactic}}}}{{{{{\mathrm{ }}}}}}\;{{{\mathrm{acid}}}}} \right]$$

The equation for J_HLac_ can be re-written:

$${{{\mathrm{J}}}}_{{{{\mathrm{HLac}}}}} =	\;{{{\mathrm{P}}}}_{{{{\mathrm{MCT}}}}} \cdot \left( {\left[ {{{{\mathrm{H}}}}^ + } \right]_{{{\mathrm{i}}}} \,\cdot\, \left[ {{{{\mathrm{lactate}}}}} \right]_{{{\mathrm{i}}}}\,-\,\left[ {{{{\mathrm{H}}}}^ + } \right]_{{{\mathrm{e}}}} \cdot \left[ {{{{\mathrm{lactate}}}}} \right]_{{{\mathrm{e}}}}} \right) \\ 	+\,{{{\mathrm{P}}}}_{{{{\mathrm{lipid}}}}} \,\cdot\, \left( {\left[ {{{{\mathrm{H}}}}^ + } \right]_{{{\mathrm{i}}}} \,\cdot\, \left[ {{{{\mathrm{lactate}}}}} \right]_{{{\mathrm{i}}}}\,-\,\left[ {{{{\mathrm{H}}}}^ + } \right]_{{{\mathrm{e}}}} \,\cdot\, \left[ {{{{\mathrm{lactate}}}}} \right]_{{{\mathrm{e}}}}} \right)/{{{\mathrm{K}}}}_{{{{\mathrm{HLac}}}}} \\ =	\;({{{\mathrm{P}}}}_{{{{\mathrm{MCT}}}}} + {{{\mathrm{P}}}}_{{{{\mathrm{lipid}}}}}/{{{\mathrm{K}}}}_{{{{\mathrm{HLac}}}}}) \cdot \left( {\left[ {{{{\mathrm{H}}}}^ + } \right]_{{{\mathrm{i}}}} \cdot \left[ {{{{\mathrm{lactate}}}}} \right]_{{{\mathrm{i}}}}\,-\,\left[ {{{{\mathrm{H}}}}^ + } \right]_{{{\mathrm{e}}}} \,\cdot\, \left[ {{{{\mathrm{lactate}}}}} \right]_{{{\mathrm{e}}}}} \right) \\ = 	\;{{{\mathrm{P}}}}_{{{{\mathrm{HLac}}}}} \cdot \left( {\left[ {{{{\mathrm{H}}}}^ + } \right]_{{{\mathrm{i}}}} \,\cdot\, \left[ {{{{\mathrm{lactate}}}}} \right]_{{{\mathrm{i}}}}\,-\,\left[ {{{{\mathrm{H}}}}^ + } \right]_{{{\mathrm{e}}}} \,\cdot\, \left[ {{{{\mathrm{lactate}}}}} \right]_{{{\mathrm{e}}}}} \right)$$

For a typical cells, ρ is ~1 µm^−1^ ([49]) and p_lipid_ is ~0.5 µm/s ([50]), thus in the absence of MCT facilitation, P_HLac_ is ~0.003 µM^−1^ s^−1^.
