# Supplementary material for: Autoregulation of H+/lactate efflux prevents monocarboxylate transport (MCT) inhibitors from reducing glycolytic lactic acid production
Source: Br J Cancer. 2022 Jul 15;127(7):1365–77. doi: 10.1038/s41416-022-01910-7 (PMC9519749; doi:10.1038/s41416-022-01910-7)
Supplement: Supplementary file 1 — Supplemental Mehtods and Supplemental Figures [file 41416_2022_1910_MOESM1_ESM.pdf]

SUPPLEMENTARY METHODS

Intrinsic buffering capacity. cSNARF1-loaded PDAC cells seeded in 4-chamber slides were superfused by a sequence of HEPES-buffered solutions containing 0 mM, 20 mM, 10 mM, 5 mM and again 0 mM NH_4Cl. Addition of NH_4Cl was osmotically compensated with a reduction in NaCl. Each solution change evokes an injection of acid due to NH_4^+ deprotonation driven by NH_3 efflux. This concentration of acid, divided by the measured pHi changes, gives buffering capacity, which was plotted as a function of mean pHi and fitted to a line[1].

Fluorescent glucose uptake assay. The pH sensitivity of the glucose uptake pathway was inferred from the rate of uptake of the fluorescent glucose derivative (2-deoxy-2-[(7-nitro-2,1,3-benzoxadiazol-4-yl) amino]-D-glucose (NBDG; N13195, ThermoFisher Scientific)[2]. Cells were first loaded with Cell Tracker Deep Red (1 μM; C34565, ThermoFisher, Scientific) to identify cell cytoplasm using a spectrally-resolvable dye. Superfusion delivered 500 μM NBDG to cells in solution titrated to a target pH. After a 15 min period of loading, during which uptake is linear and not yet saturated, extracellular dye was washed away with NDBG-free solution and multiple fields of view were imaged within 2 min to quantify dye uptake (excitation 488 nm, emission 520 nm) in Cell Tracker Deep Red positive regions (excitation 633 nm, emission >650 nm).

Well-based fluorimetric assay of lactic acid production and respiratory rate. This dual-dye fluorimetric assay was based on a recently published method[3]. Cells were plated in a 96-well plate at 70k/per well. Media were buffered with various combinations of HEPES/MES or CO_2/HCO_3^-. Note that media buffered with the latter will show a transient pHe behaviour as CO_2 from the atmosphere dissolves into the HCO_3^- containing medium. pH was measured using HTPS (2 μM) and oxygen tension was inferred from RuBPY (50 μM). Prior to measurements, media were covered with a layer of 150 μL mineral oil (M5904, Sigma) to impose a barrier to gas diffusion. Cumulative H^+ production and O_2 consumption were measured according to equations described previously [3]. The plate was placed immediately in a Biotek Cytation 5 plate reader pre-heated to 37°C, recording HPTS and RuBPY fluorescence sequentially.

Six-state mathematical model of MCT. MCT-dependent H^+-flux data were fitted to a six-state model[4]. Under the experimental conditions, the model was reduced to three variables: the number of MCT transporters (related to protein abundance at the membrane), a rate constant (k_b) describing the deprotonation of an exofacial state of MCT, and an equilibrium constant (K_2) describing the equilibrium between intra- and extracellular facing MCT bearing no ligands. All other variables were constrained thermodynamically, or assumed to have diffusion-limited values, such as the rate of protonation. The ratio of protonation to deprotonation rate constants informs about the apparent pK_a of the relevant binding site.

Additional references

1 Leem, C. H., Lagadic-Gossmann, D. & Vaughan-Jones, R. D. Characterization of intracellular pH regulation in the guinea-pig ventricular myocyte. *J Physiol* **517 (Pt 1)**, 159-180 (1999).

2 Yamada, K., Saito, M., Matsuoka, H. & Inagaki, N. A real-time method of imaging glucose uptake in single, living mammalian cells. *Nat Protoc* **2**, 753-762 (2007).

3 Blaszczak, W., Tan, Z. C. & Swietach, P. Cost-Effective Real-Time Metabolic Profiling of Cancer Cell Lines for Plate-Based Assays. *Chemosensors* **9** (2021).

4 Almquist, J. *et al.* A kinetic model of the monocarboxylate transporter MCT1 and its interaction with carbonic anhydrase II. *Journal of Computer Science & Systems Biology* **3** (2010).

SUPPLEMENTARY FIGURES

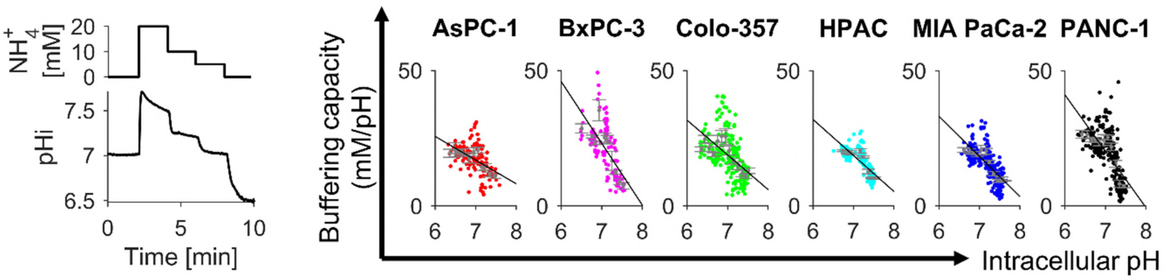

Figure S1: Protocol for measuring intrinsic buffering capacity (left) and data from 6 PDAC lines, expressed as a function of pHi and fitted to a line (grey); n=300-500/N=3.

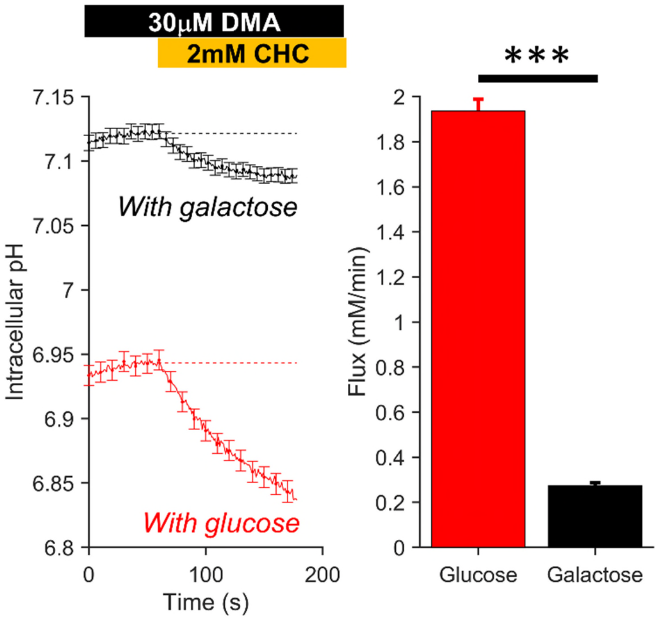

Figure S2: Acute exposure of MIA Paca2 cells to 2mM CHC under conditions that block pH regulation (HEPES-buffered superfusates containing 30 μM dimethylamioride). Intracellular pH measured by cSNARF1 loaded into cells (glucose: mean of 408 cells from 8 loading; galactose: mean of 727 cells from 6 loadings). Mean±SEM. If the metabolic substrate is glucose, CHC produces a robust acidification arising from the cytoplasmic retention of lactic acid produced glycolytically. If the metabolic substrate is replaced with galactose (that in culture does not yield substantial quantities of lactic acid), the effect of CHC is significantly reduced (by a factor of 10).

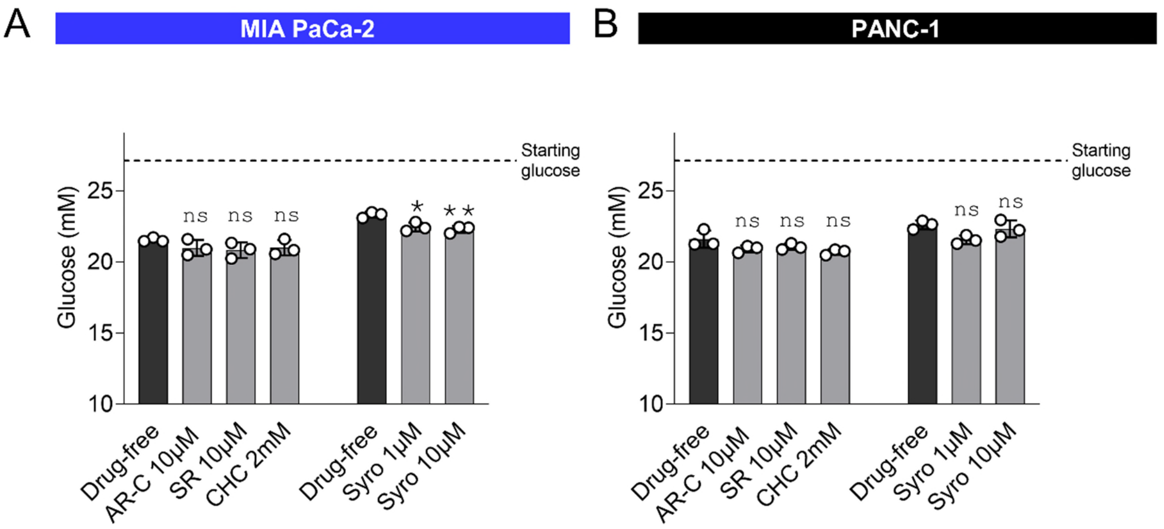

Figure S3: End-point glucose concentration, paired with lactate measurements in Figure 3D. Dashed line shows medium glucose measured in cell-free cells. Difference indicates glucose consumption through all relevant metabolic pathways The data are plotted as mean ± SD.

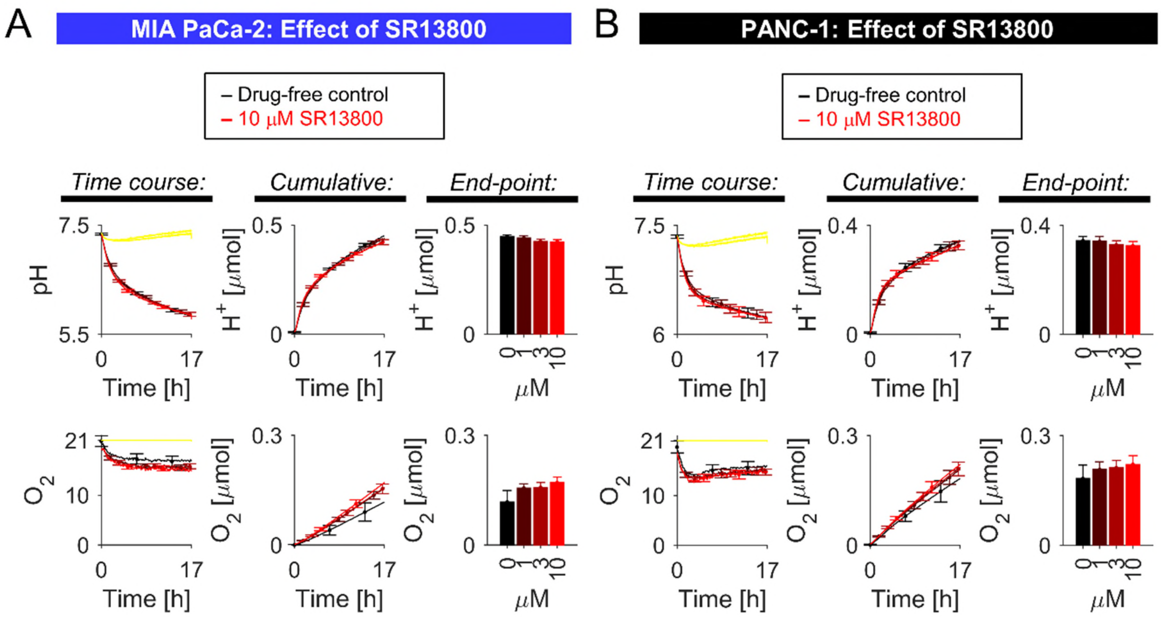

Figure S4: A. Fluorimetric assay for acid production and oxygen consumption performed in media buffered with 2 mM HEPES and 2 mM MES, or a formulation that was supplemented with 30 mM HEPES to raise buffering power. Augmented buffering accelerates glycolytic rate to an extent that also reduces the cell's reliance on mitochondrial respiration. Experiments performed on MIA PaCa-2 and PANC-1 cells (n=12/N=4). Significant effect of buffer augmentation on glycolytic rate ($P<0.01$) in both cell lines and respiratory rate ($P<0.01$ for MIA PaCa2- and $P<0.05$ for PANC-1); two-way ANOVA. B. Fluorimetric assay for acid production and oxygen consumption performed on MIA PaCa-2 cells in presence of SR13800 (n=8); B. experiment in PANC-1 cells (n=8). No significant effect of drug relative to paired controls (repeated measures ANOVA).

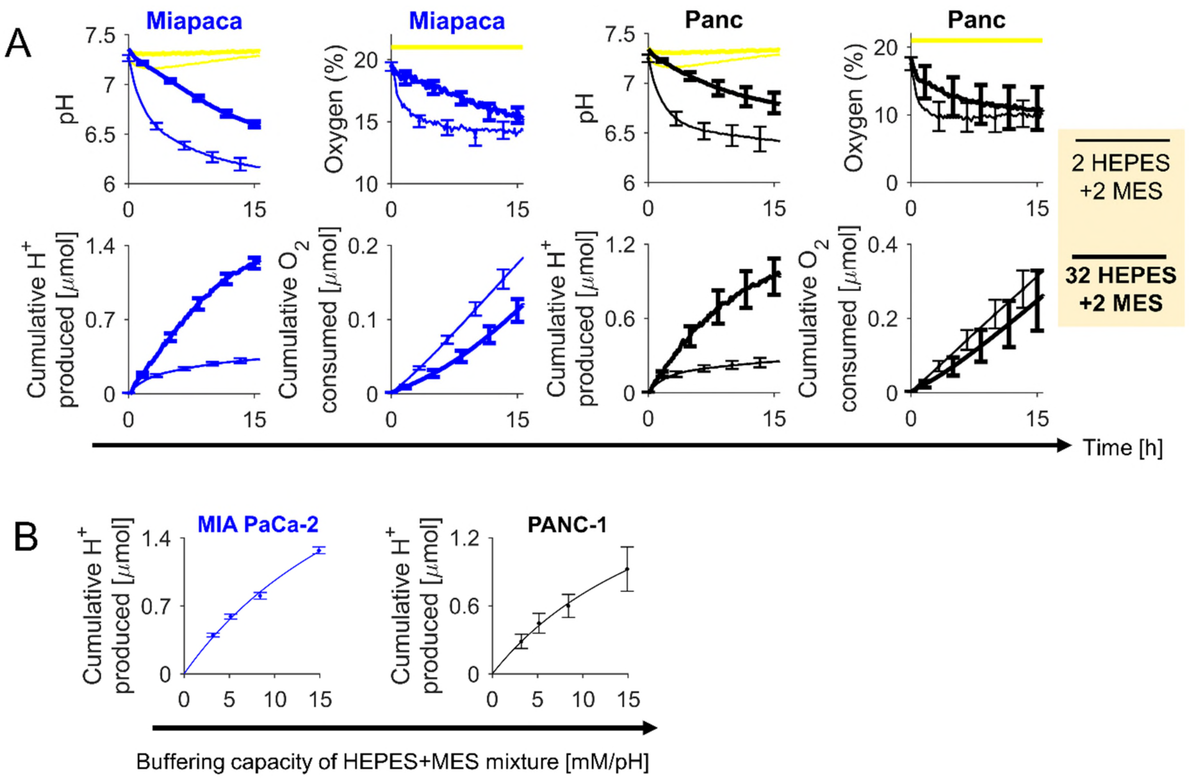

Figure S5: A. Relationship between cumulative acid production and buffering power varied by raising HEPES and MES concentration (2+2, 5+5, 10+10 and 20+20 mM). Experimental performed on MIA PaCa-2 and PANC-1 cells (n=12/N=4). All changes to buffering were osmotically compensated with NaCl.

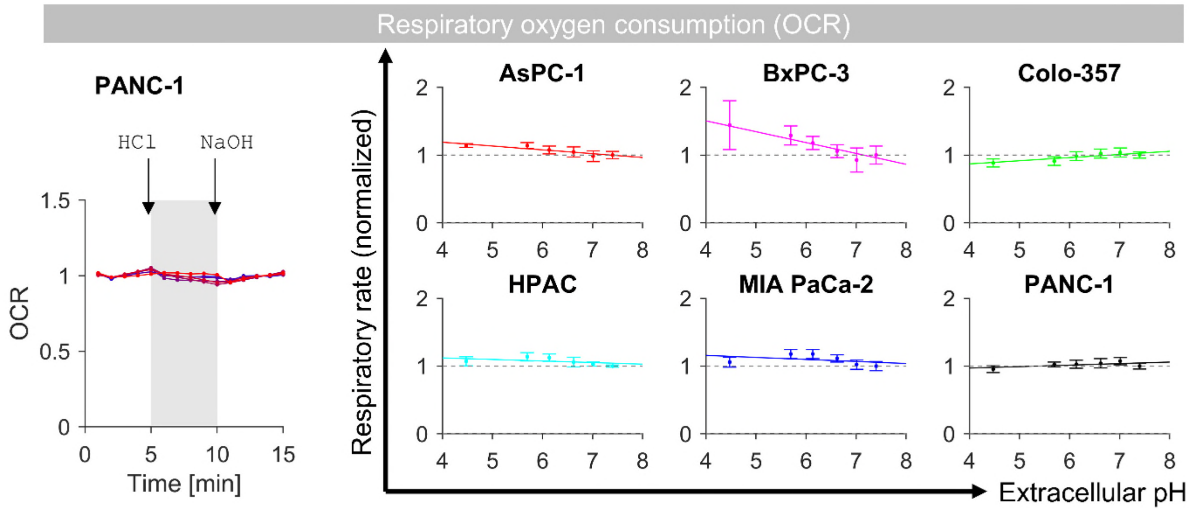

Figure S6: Oxygen consumption rate is insensitive to extracellular pH. Measurements of ECAR were paired with measurements of oxygen consumption rate (OCR), a measure of respiration. Unlike ECAR, OCR was pH-insensitive. pH-sensitivity determined from response to reducing medium pH from 7.4 with HCl injection, and then restoring pH to 7.4 with matching NaOH injection (n=8/N=2). Grey lines show pHe-ECAR curve averaged for all 6 lines.

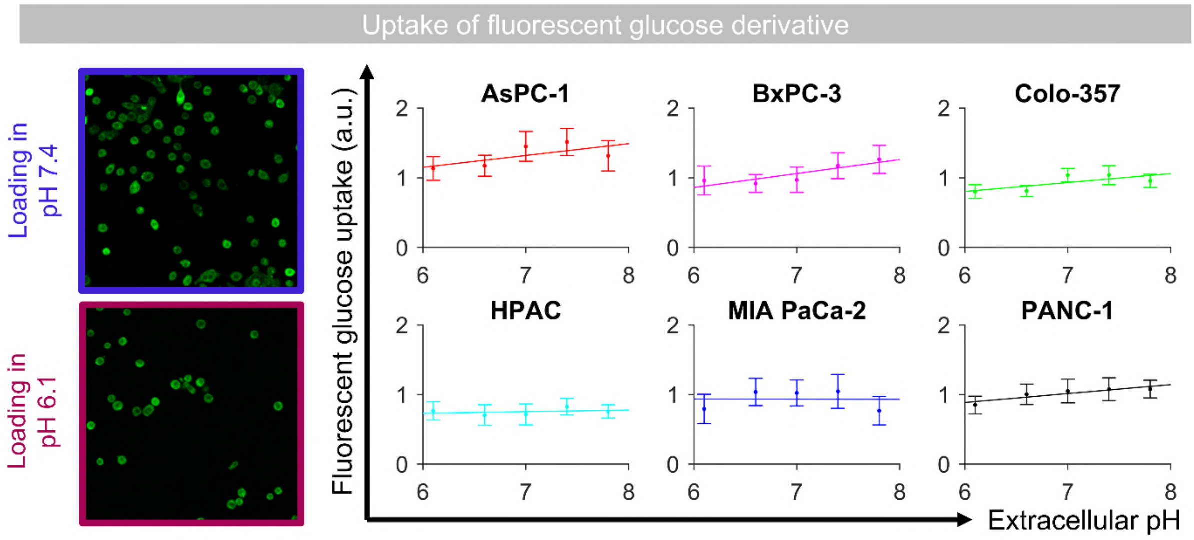

Figure S7: Measuring the pH-sensitivity of glucose uptake pathways. Cells exposed to solution containing fluorescent glucose derivative NBDG for 15 min at a test pH. After the loading period, cells were rapidly washed by superfusion in dye-free solution. Images were quantified for cellular NBDG uptake within cytoplasmic regions, indicated with CellTracker DeepRed. NBDG uptake was determined to be pH-insensitive.

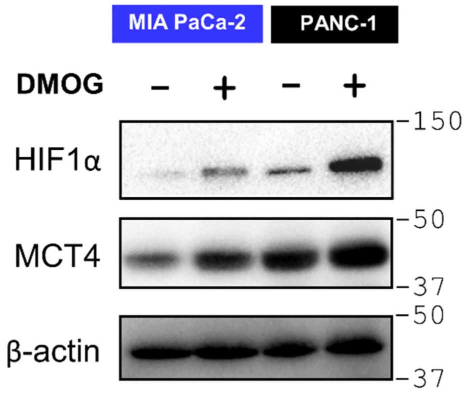

Figure S8: Western blot showing stabilization of HIF-dependent hypoxic signaling in response to 48 h treatment with 1 mM DMOG.

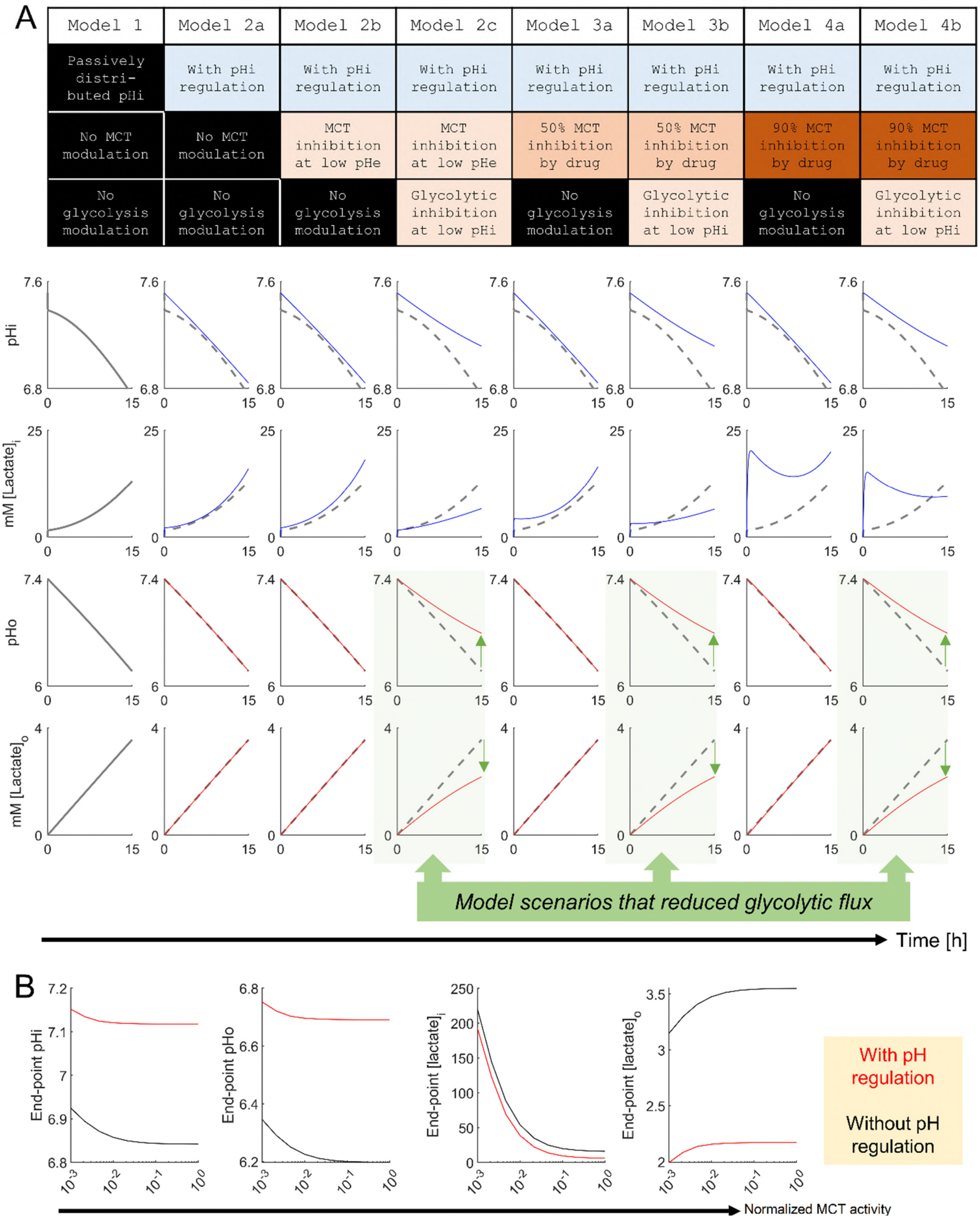

Figure S9: Mathematical modelling supports the notion that glycolytic rate is not set by MCT permeability. A. Model of a glycolytic PDAC cells was parameterized on the basis of experimental conditions and data averaged across the six PDAC lines. The relevant parameters included: medium volume (100 µL) and buffering capacity (2 mM HEPES, 2 mM MES), intrinsic buffering capacity (Fig S1), cellular glycolytic rate (Fig 2B) and its pHi sensitivity (Fig 6D), and P_{MCT} and is pHe-sensitivity (Fig 5A). Models included literature values for cell volume (2000 fL) assuming spherical symmetry

(radius 9.8 μm, surface area/volume ratio of 3/radius), lactic acid permeability across the lipid bilayer of 0.5 μm/s, and assumed 100,000 cells per well. Eight different models were run to simulate one of eight conditions, as described in the top panel. Model 1 was the baseline scenario and featured no pHi regulation (i.e. allowed pHi to change according to glycolytic production and venting), had no pHe sensitivity of MCT or pHi-sensitivity of glycolysis. The results of this model (grey) were used as a reference for other model outputs (replicated as grey dashed lines). Model 2a included pHi regulation, which was modelled to couple pHi to pHe according to the pHe-pHi relationship (Fig 6B). Model 2b was based on Model 2a and included pHe-sensitivity of P_{MCT} (Fig 5A). Model 2c was based on Model 2b and included pHi-sensitivity of glycolysis (Fig 6D). Model 3a was based on Model 2a but with a 50% reduction in P_{MCT}. Model 3b was based on Model 3a but included pHi-sensitive glycolysis. Model 4a was based on Model 2a but had a 90% reduction in P_{MCT}. Model 4b was based on Model 2a but included pHi-sensitive glycolysis. The models simulated intracellular pH and [lactate] (top panels, blue) and extracellular pH and [lactate] (bottom panels, red). The bottom panels are a readout of glycolytic output; relative to Model 1, this was reduced only with Models 2c, 3b and 4b. The component that is common to these three models is a pHi-sensitivity of glycolysis, indicating this to be the major physiological controller of fermentative metabolism. The results also indicate that changes in P_{MCT}, evoked by pHe (Models 2b/c) or drugs (Models 3a/b and 4a/b), do not directly reduce glycolytic rate. B. Model results at the end of a 15 h simulation period performed over a range of P_{MCT} activity (presented as normalized to control levels). Models were based on a version of Model 2c with or without pHi regulation (red/black, respectively). P_{MCT} was reduced by up to 1000-fold. Note that glycolytic output, gauged by end-point extracellular [lactate], only began to decrease with MCT inhibition greater than 100-fold.
